# Vitamins and Human Health: 2nd Edition Editorial

**DOI:** 10.3390/nu17091534

**Published:** 2025-04-30

**Authors:** Tyler Barker

**Affiliations:** 1 Sports Medicine Research Institute, College of Medicine, The Ohio State University Wexner Medical Center, Columbus, OH 43202, USA; tyler.barker@osumc.edu; 2 Department of Orthopaedics, University of Utah, Salt Lake City, UT 84108, USA

Vitamins are essential compounds fundamental to maintaining health; inadequate intake or endogenous deficiencies are identified as risk factors for various disabilities, injuries, illnesses, impaired rehabilitation, and diseases [1,2,3,4]. This second-edition Special Issue features reviews and original research articles regarding vitamins in health and disease. Research investigating the association of vitamins with systemic inflammation, pulmonary function, chronic obstructive pulmonary disease (COPD), and diabetes and studies on the antioxidative properties of vitamin D that regulate skeletal muscle size and function, as well as many more topics, are included in this Special Issue. The studies contributing to this Special Issue collectively highlight the multi- and transdisciplinary nature of vitamins in health and disease. Below is a brief summary of the articles included in this Special Issue’s second edition.

Vitamins A and C

Vitamins possess diverse anti-inflammatory properties. Circulating high-sensitivity C-reactive protein (hsCRP) is an established biomarker of systemic inflammation that is routinely integrated into clinical practice and research. In a unique investigation, Baik [contribution 1] examined the association between dietary vitamin intake and the risk of elevated serum hsCRP concentration. This study considered a serum hsCRP concentration > 3.0 mg/L as an indicator of systemic inflammation, and vitamin intake was assessed using a food frequency questionnaire. The results illustrated an inverse association between vitamin C intake and an elevated hsCRP concentration, while β-carotene intake exhibited a U-shaped association. These associations were exaggerated in individuals with established risk factors. From these data, Baik concluded that the dietary intake of vitamins A and C attenuated systemic inflammation in adults with and without additional risk factors.

Vitamins A and E

In a study utilizing Korea National Health and Nutrition Examination Survey (KNHANES, 7th survey, 2016–2018) data, Noh and Baik [contribution 2] investigated the association of serum vitamin A (retinol) and vitamin E (α-tocopherol) concentrations with pulmonary function and COPD. This cross-sectional study found a positive association between serum vitamin A concentrations and the forced expiratory volume. Serum vitamin A concentrations were also inversely related to the COPD prevalence. The findings related to vitamin A, however, could be influenced by systemic inflammation, as the results were limited to individuals with a serum C-reactive protein concentration ≤ 1 mg/L. Surprisingly, the serum vitamin E concentrations were not associated with pulmonary function or COPD prevalence. Additional research examining the mechanisms governed by vitamins A and E in pulmonary function and COPD is desired.

B Vitamins

B vitamins consist of eight water-soluble vitamins [contribution 3]. Homocysteine is derived from methionine via the vitamin B_6_ and B_12_ pathways, and plasma homocysteine levels are reduced through B vitamin supplementation. Although lower plasma homocysteine levels have been found to be associated with a decreased risk of cardiovascular-related events, the results are inconclusive regarding the association of B vitamin supplementation with thromboembolic events. Li and colleagues [contribution 3] conducted a review of randomized controlled trials assessing the role of B vitamin supplementation on thrombotic risk reduction. From this review, few studies were identified and conflicting results were found regarding the role of B vitamin supplementation in reducing thrombotic risk. Li and colleagues [contribution 3] suggested that more clinical trials and translational studies are needed to further delineate the relationship between B vitamin supplementation and thrombosis risk.

Vitamin D

Vitamin D is a pleiotropic vitamin essential to calcium and phosphate homeostasis. While its anti-inflammatory properties have been and continue to be extensively studied, research over the past few decades has revealed a potential antioxidant role of vitamin D. In addition, widespread efforts suggest a regulatory influence of vitamin D on skeletal muscle size and function. In an eloquent and thorough review article, Russo and colleagues [contribution 4] provided a detailed description of the biological and molecular effects of vitamin D on skeletal muscle and the antioxidative influence of vitamin D on skeletal muscle dysfunction. Russo and colleagues [contribution 4] also identified future research opportunities regarding the antioxidant, anti-inflammatory, and neuroprotective properties of vitamin D on skeletal muscle.

Bae and colleagues [contribution 5] investigated the association of vitamin D deficiency with knee osteoarthritis diagnosis following anterior cruciate ligament reconstruction (ACLR). In a retrospective study, patients who underwent ACLR were classified as vitamin D-deficient (serum 25(OH)D ≤ 20 ng/mL) or non-deficient (serum 25(OH)D > 20 ng/mL). Vitamin D deficiency was found to be associated with an increased occurrence of and a shortened time to a knee osteoarthritis diagnosis following ACLR. This finding potentially implies that a low serum 25(OH)D concentration may serve as a prognostic biomarker for identifying patients at risk of developing a chronic, degenerative disease following one of the most commonly injured and surgically repaired ligaments in the knee [contribution 5].

In a quasi-randomized study, Savolainen and colleagues [contribution 6] studied the influence of supplemental vitamin D (8000 IU/day of cholecalciferol) during a 12-week supervised resistance training program on skeletal muscle strength, lean body mass, and cardiorespiratory fitness (i.e., VO_2_max) compared to a placebo. The male participants were middle-aged (>50 years), reportedly healthy, and predominately vitamin D-insufficient (serum 25(OH)D < 30 ng/mL) prior to supplementation and the initiation of the resistance training program. In this investigation, it was seen that supplemental vitamin D increased the serum 25(OH)D without augmenting strength gains, increasing lean body mass, or decreasing fat mass compared to the placebo group following the resistance training program. The cardiorespiratory fitness was not significantly altered in the supplemental vitamin D and placebo groups. Based on these results, Savolainen and colleagues [contribution 6] concluded that supplemental vitamin D does not appear to improve strength gains or body composition alterations induced by a supervised resistance training program despite increasing serum 25(OH)D in reportedly healthy men with an initially low serum 25(OH)D.

Using data from the National Health and Nutrition Examination Survey (NHANES) 2011–2016, Hung and colleagues [contribution 7] examined the association between vitamin D and dental caries. After accounting for sociodemographic factors and body mass index, data from this investigation highlighted a strong relationship between a low serum 25(OH)D concentration and an increased risk of dental caries [contribution 7]. The findings by Hung and colleagues [contribution 7] emphasize that individuals with an insufficient serum 25(OH)D concentration are challenged by an increased likelihood of dental caries and underscore the importance of proactively achieving and maintaining an adequate serum 25(OH)D concentration for optimal dental health.

In this Special Issue, an article by Kowalcze and colleagues [contribution 8] assessed the role of low gestational vitamin D status on minipuberty, a term describing the temporary, sex-specific activation of the hypothalamic–pituitary–gonadal axis soon after birth. This study included infant daughters of women with deficient, insufficient, and normal serum 25(OH)D concentrations. Urine and saliva samples were periodically collected during the first 18 months of life. To summarize the novel findings, a low vitamin D status during gestation resulted in a more pronounced and persistent activation of the reproductive axis and was associated with increased dimensions of sexual organs [contribution 8].

Vitamin D deficiency is common and associated with complications, impairments, and prolonged hospital stays in intensive care unit (ICU) patients. Wang and colleagues [contribution 9] reviewed recent randomized controlled trials examining the impact of supplemental vitamin D on outcomes in critically ill adult patients. Vitamin D supplementation was found to be associated with a shortened ICU or hospital length of stay, improved Sequential Organ Failure Assessment scores, and a reduced duration of mechanical ventilation in critically ill patients, while evidence linking supplemental vitamin D to a lower 28-day mortality was uncertain [contribution 9]. Wang and colleagues [contribution 9] suggested that many factors should be considered when determining the use of supplemental vitamin D in critically ill patients.

Depression is a leading cause of disease burden worldwide and a debilitating mental disorder that diminishes the quality of life. Seasonal affective disorder can be described as a recurrent form of depression, with annual episodes varying in severity. In a review article, Jahan-Mihan and colleagues [contribution 10] explored the literature regarding the role of dietary and supplemental vitamins in the prevention and treatment of depression and seasonal affective disorder in adults. Evidence was found to support the role of vitamins C and D and B vitamins in preventing and treating depression, but additional research is required to determine efficient and effective interventional strategies and to identify the potential mechanisms of action targeted by vitamins [contribution 10].

Vitamin K

Vitamin K is a fat-soluble micronutrient traditionally recognized for its regulatory role in blood coagulation and as an anti-inflammatory and antioxidant agent. Compelling evidence also identifies the potential role of vitamin K in glucose homeostasis. With this role in mind, De La Barrera and colleagues [contribution 11] investigated the causal association between vitamin K1 (phylloquinone) and type 1 diabetes using a two-sample Mendelian randomization approach. De La Barrera and colleagues [contribution 11] also implemented a multivariable Mendelian randomization design to examine the potential effect of gut microbiome populations on the association between vitamin K and type 1 diabetes. The results from this study suggested that the serum vitamin K1 levels did not have a large causal effect on the risk of type 1 diabetes. However, integrating gut microbiome populations into the analysis identified a suggestive causal effect of vitamin K on the risk of type 1 diabetes [contribution 11]. However, and as stated by the authors [contribution 11], their results should be interpreted with caution and warrant additional investigation.

In adolescent and adult patients with cystic fibrosis, Krzyzanowska-Jankowska and colleagues [contribution 12] studied the vitamin K status based on the serum K1, menaquinone (MK)-4, and MK-7 concentrations, which were compared to those of healthy controls. In the absence of vitamin K1 supplementation, the serum vitamin K1 concentrations were not significantly different between the groups, while the MK-4 concentrations were higher and the MK-7 concentrations were lower in the cystic fibrosis patients compared to those in the healthy controls. The cystic fibrosis patients that were supplemented with K1 and MK-7 displayed higher MK-7 concentrations than those receiving vitamin K1 alone or no supplement. The authors concluded that vitamin K1 concentrations were low in cystic fibrosis if not supplemented, and, in comparison to healthy controls, the MK-4 concentrations were higher in cystic fibrosis patients supplemented with large doses of vitamin K1 [contribution 12].

In summary, the articles in this second-edition Special Issue underscore the importance of vitamins in health and disease and identify future research opportunities in this area. Future research will continue to advance our knowledge of vitamins in health and disease along with their societal and clinical impacts.
